# Induction versus adjuvant chemotherapy combined with concurrent chemoradiotherapy in locoregionally advanced nasopharyngeal carcinoma: a retrospective cohort study

**DOI:** 10.18632/aging.204246

**Published:** 2022-08-26

**Authors:** Xiaoli Mu, Hongyan Liu, Juan Wu, Shi Chen, Xingchen Peng, Jingjing Wang, Zhigong Wei, Ling He, Jiyan Liu, Zejun Lu, Yonglin Su

**Affiliations:** 1 The Department of Biotherapy, Cancer Center, West China Hospital, Sichuan University, Chengdu 610041, Sichuan, China; 2 Department of Oncology, Fuyang Cancer Hospital, Fuyang 236000, Anhui, China; 3 Out-Patient Department, West China Hospital, Sichuan University, Chengdu 610041, Sichuan, China; 4 Department of Oncology, Chengdu Jinniu District People’s Hospital, Chengdu 610041, Sichuan, China; 5 Senior Department of Oncology, The Fifth Medical Center of PLA General Hospital, Beijing 100039, China

**Keywords:** locoregionally advanced nasopharyngeal carcinoma, additional chemotherapy, concurrent chemoradiotherapy, efficiency, toxicities

## Abstract

Background: Currently available evidence favors the combination of chemotherapy with concurrent chemoradiotherapy in locoregionally advanced nasopharyngeal carcinoma (LANPC). However, the optimal timing for additional chemotherapy is unclear. This study was conducted to compare the efficacy and toxicity of induction chemotherapy plus concurrent chemoradiotherapy (IC+CCRT) versus concurrent chemoradiotherapy plus adjuvant chemotherapy (CCRT+AC).

Methods: Two medical centers in China enrolled patients with LANPC (stage III-IVB) between January 2009 and May 2020. Through the use of propensity score matching (PSM), baseline characteristics were balanced. The primary endpoint was overall survival (OS), which was evaluated by the Kaplan-Meier method and log-rank test. Potential independent prognostic factors were identified using univariate and multivariate Cox proportional hazard analyses. Based on the chi-squared test, we compared the adverse events associated with treatment between the groups.

Results: After the implementation of PSM, 159 patients treated with IC+CCRT and 72 patients treated with CCRT+AC were eventually enrolled in this study. There was no significant difference between patients treated with IC+CCRT and CCRT+AC in terms of 3-year OS (94.7% versus 90.9%, p=0.816), progression-free survival (PFS) (91.2% versus 83.1%, p=0.588), locoregional recurrence-free survival (LRFS) (92.5% versus 81.8%, p=0.478), or distant metastasis-free survival (DMFS) (93.4% versus 88.2%, p=0.783). There was no prognostic significance of the treatment for OS, PFS, LRFS, or DMFS (all p > 0.05) in the univariate and multivariate analyses. Patients treated with CCRT+AC had a higher incidence of grade 3 to 4 leucopenia (p=0.001) and neutropenia (p=0.001) than those treated with IC+CCRT.

Conclusions: IC plus CCRT achieved comparable survival outcomes to CCRT plus AC and had a lower incidence of toxicity.

## INTRODUCTION

Nasopharyngeal carcinoma (NPC) is a type of epithelial malignancy arising from the nasopharyngeal mucosa, most of which occurs in the top and lateral walls of the nasopharynx, especially in the pharyngeal recess [1]. NPC's global geographical distribution is highly asymmetric. There were 129000 new cases of NPC reported in 2018. However, over 70% of new cases were found in East and Southeast Asia [2, 3]. The uneven distribution is mainly related to Epstein–Barr virus infection and genetic and environmental factors [4, 5].

Due to the widespread use of precision radiotherapy techniques such as intensity-modulated radiation therapy (IMRT) and comprehensive radiotherapy and chemotherapy, local control rates of NPC have reached 90% or higher [6]. Nevertheless, early symptoms of NPC are not typical; more than 60% of patients have developed locally advanced or late disease at the time of diagnosis [7]. For patients with LANPC, concurrent chemoradiotherapy (CCRT) with adjuvant chemotherapy (AC) and induction chemotherapy (IC) followed by CCRT are both recommended options in the latest National Comprehensive Cancer Network (NCCN) guidelines [8]. The intergroup trial 0099 demonstrated for the first time that CCRT followed by AC resulted in a survival benefit compared to RT alone. Since then, three cycles of cisplatin concurrent chemoradiotherapy and three cycles of cisplatin plus five fluorouracil (PF) adjuvant chemotherapy have become the standard treatment for LANPC in North America [9]. Ribassin-Majed et al. enrolled 4,806 patients in 19 clinical trials to systematically assess the most effective treatment for LANPC, and the results indicated that adding AC to chemotherapy improved PFS compared to chemotherapy alone (HR=0.81; 95% CI: 0.66-0.98) [10]. However, the majority of patients are unable to withstand the side effects of AC because of the significant toxicity during CCRT, which restricts the widespread use of AC. IC is also a therapy option for LANPC patients, which is performed prior to CCRT and has better patient compliance. In a phase III randomized controlled trial conducted by Sun Yat-sen University, patients who received IC plus CCRT showed a significant survival advantage over those who received CCRT alone, indicating that the combination of IC with CCRT is effective in enhancing survival in LANPC [11].

Despite the fact that researchers have analyzed and evaluated additional chemotherapy, such as AC and IC, in clinical trials, whether to give these patients chemotherapy before or after systemic therapy/RT is still unclear and debatable. Therefore, this study evaluated and compared the efficacy and toxicity of IC+CCRT and CCRT+AC for LANPC patients in the real world.

## RESULTS

### Study subjects

From January 2009 to May 2020, 463 LANPC patients with complete and detailed clinical information were included in the study. Among them, 389 LANPC patients treated with IC+CCRT were from West China Hospital of Sichuan University, and 74 patients receiving CCRT+AC were from the People's Liberation Army General Hospital. Since subjects were not randomly assigned to either treatment modality, it can be assumed that certain preexisting factors influenced their efficacy and status. Therefore, PSM analysis was used to match baseline characteristics between the two groups and to form a single participant group with similar baseline characteristics. Patients in the IC+CCRT group were systematically matched to patients in the CCRT+AC group with the closest propensity score. The jitter plots and the histograms of propensity scores are shown in Supplementary data (Supplementary Figure 1).

### Patient characteristics

Before PSM, the median follow-up time of the 463 patients was 55 months (range 6-115 months). The majority of patients in both groups were middle-aged men; patients with T3-T4 stage accounted for 72.5%, and 70.7% of patients were diagnosed as clinical stage IVa/IVb in the IC+CCRT group, while in the CCRT+AC group, more than half of the patients were diagnosed as stage III. After the implementation of PSM, 231 patients were successfully matched with 5 balanced covariates. The median follow-up time was 56 months (range 8-115). In this period, 26/159 (16.4%) patients died in the IC+CCRT group, and 14/72 (19.4%) patients died in the CCRT+AC group. The baseline characteristics are shown in Table 1.

**Table 1:** Characteristics of patients at baseline before and after PSM.

**Characteristics**	**Before PSM**			**After PSM**		
	**IC+CCRT N=389**	**CCRT+AC N=74**	P value^*^	**IC+CCRT N=159**	**CCRT+AC N=72**	P value^*^
Age						
<50	237(60.9%)	38(51.3%)	0.1242	88(55.3%)	38(52.8%)	0.7165
≥50	152(39.1%)	36(48.7%)		71(44.7%)	34(47.2%)	
Sex						
Male	288(74.0%)	52(70.3%)	0.5014	113(71.1%)	51(70.8%)	0.9708
Female	101(26.0%)	22(29.7%)		46(28.9%)	21(29.2%)	
T classification						
T1-2	107(27.5%)	29(39.2%)	0.0431	41(25.8%)	27(37.5%)	0.0704
T3-4	282(72.5%)	45(60.8%)		118(74.2%)	45(62.5%)	
N classification						
N0-1	88(22.6%)	9(12.2%)	0.0427	27(17.0%)	9(12.5%)	0.3844
N2-3	301(77.4%)	65(87.8%)		132(83.0%)	63(87.5%)	
Stage						
III	114(29.3%)	55(74.3%)	0.0001	102(64.2%)	53(73.6%)	0.1564
IVa/IVb	275(70.7%)	19(25.7%)		57(35.8%)	19(26.4%)	
IC/AC regime			/			/
TPF	261(67.1%)	0		109(68.6%)	0	
GP	76(19.5%)	0		29(18.2%)	0	
TP	11(2.8%)	55(74.3%)		6(3.8%)	54(75.0%)	
PF	25(6.4%)	15(20.3%)		11(6.9%)	14(19.4%)	
Other	16(4.1%)	4(5.4%)		4(2.5%)	4(5.6%)	

^*^P>0.05.

### Survival outcomes

**Figure 1:**
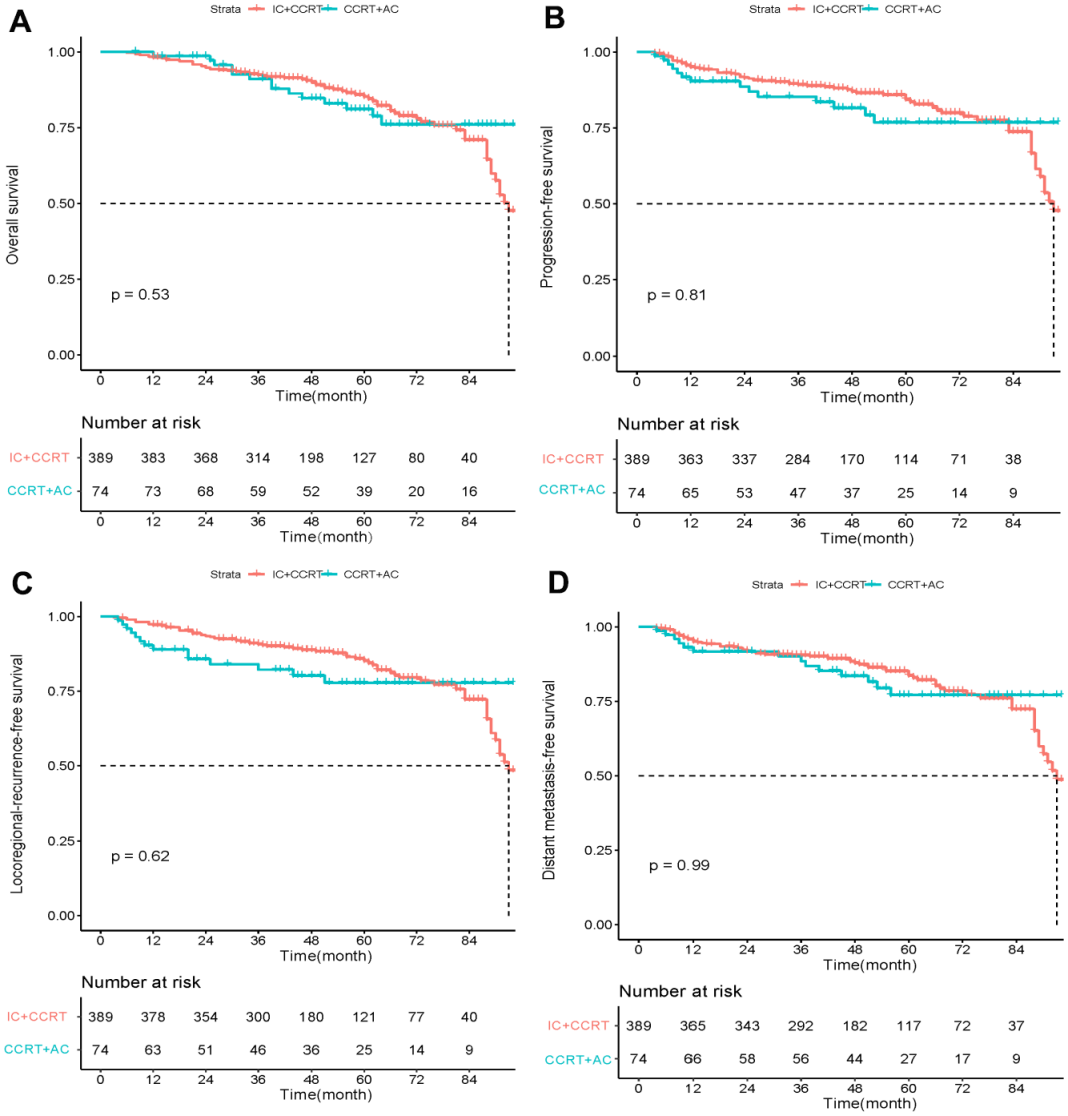
Kaplan–Meier curves of overall survival (**A**), progression-free survival (**B**), locoregional recurrence-free survival (**C**), and distant metastasis-free survival (**D**) in LANPC patients treated with IC+CCRT or CCRT+AC before PSM.

**Figure 2:**
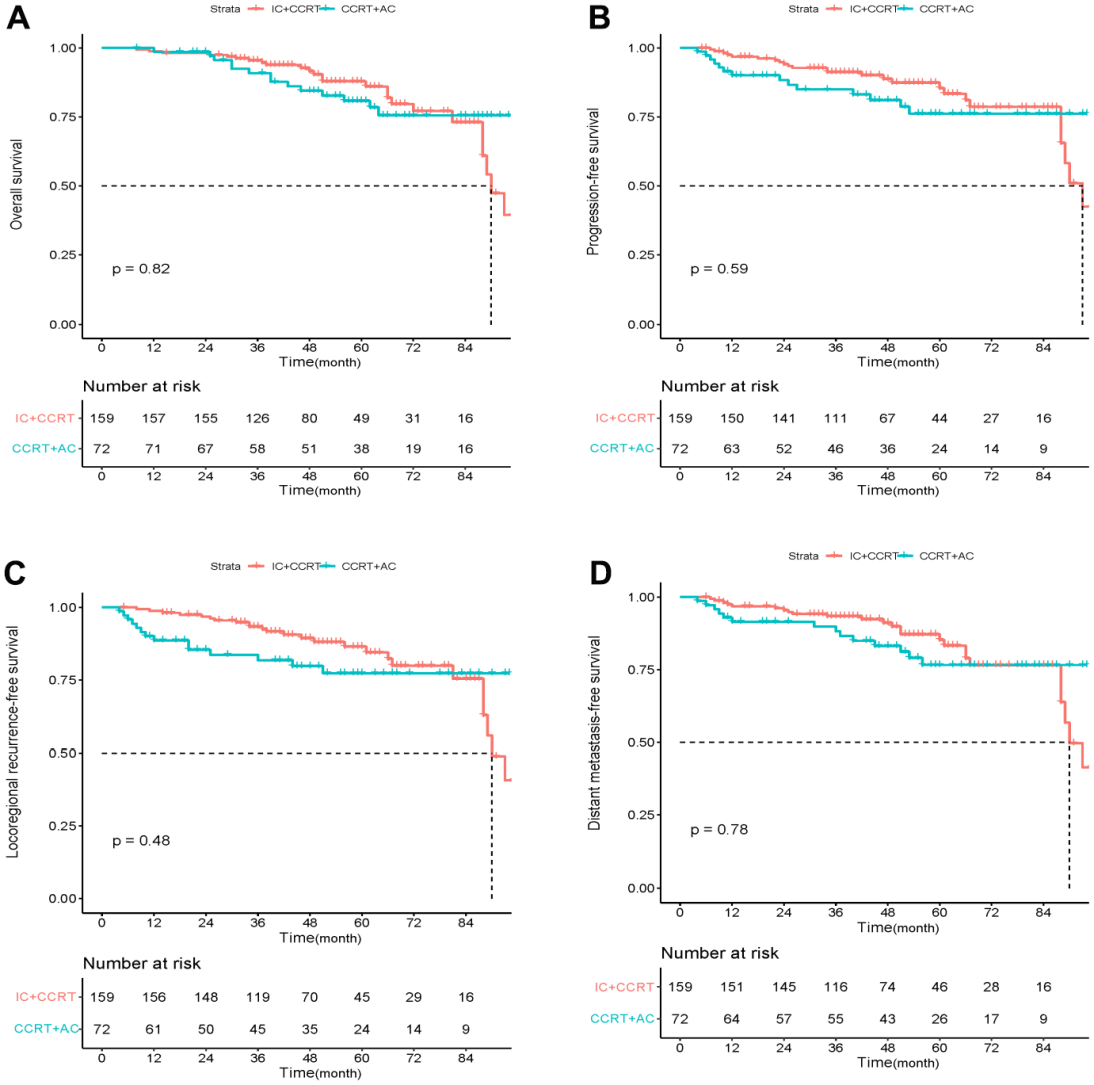
Kaplan–Meier curves of overall survival (**A**), progression-free survival (**B**), locoregional recurrence-free survival (**C**), and distant metastasis-free survival (**D**) in LANPC patients treated with IC+CCRT or CCRT+AC after PSM.

Before PSM, the 3- and 5-year OS rates of IC+ CCRT were 92.4% and 85.2%, and those of the CCRT+AC group were 91% and 78.8%, respectively. After PSM, the 3- and 5-year OS rates for IC+ CCRT were 94.7% and 86.0%, respectively, while they were 90.9% and 78.2% for the CCRT+AC group. However, as illustrated in Table 2, before or after PSM, there was no discernible difference in OS between the two groups at 3 and 5 years. Kaplan-Meier curves also showed no significant difference in OS, PFS, LRFS, or DMFS between the IC+CCRT and CCRT+ AC groups before or after PSM (Figures 1, 2).

**Table 2:** Patient survival (%) at 3 years and 5 years before and after PSM.

**Survival outcomes**	**Before PSM**			**After PSM**		
	**IC+CCRT N=389**	**CCRT+AC N=74**	**P value^†^**	**IC+CCRT N=159**	**CCRT+AC N=72**	**P value**
**OS**			*0.530*			*0.816*
At 3-year	92.4%	91.0%		94.7%	90.9%	
At 5-year	85.2%	78.8%		86.0%	78.2%	
**PFS**			*0.805*			*0.588*
At 3-year	89.2%	83.5%		91.2%	83.1%	
At 5-year	84.4%	76.8%		85.4%	76.2%	
**LRFS**			*0.620*			*0.478*
At 3-year	90.7%	82.2%		92.5%	81.8%	
At 5-year	85.2%	77.8%		84.5%	77.3%	
**DMFS**			*0.988*			*0.783*
At 3-year	90.5%	88.5%		93.4%	88.2%	
At 5-year	83.7%	77.1%		85.2%	76.6%	

^†^P<0.05.

### Prognostic analysis

A Cox proportional hazards model was utilized to investigate each variable's power for OS, PFS, LRFS, and DMFS. The tested factors included age, sex, T stage, N stage, clinical stage, and treatment regimen. Nevertheless, the elements included in this study did not indicate any correlation with OS, PFS, LRFS, or DMFS (all p> 0.05); the treatment regimen was no exception (Table 3).

**Table 3:** Univariate and multivariate analysis of prognostic factors in LANPC patients.

	**Univariate analysis**			**Multivariate analysis**		
	**HR**	**95%CI**	**P value**	**HR**	**95%CI**	**P value^‡^**
**OS**						
**Treatment Group** CCRT+AC vs IC+ CCRT	0.925	0.477-1.793	0.816	0.887	0.452-1.740	0.726
**Age** ≥50 vs <50	1.462	0.785-2.722	0.232	1.440	0.738-2.810	0.285
**Sex** Male vs Female	0.573	0.297-1.108	0.098	0.519	0.264-1.020	0.057
**T classification** T3-4 vs T1-2	0.847	0.444-1.616	0.614	0.670	0.320-1.402	0.287
**N classification** N2-3 vs N0-1	0.660	0.300-1.449	0.300	0.604	0.241-1.512	0.282
**Stage** IVa/IVb vs III	1.121	0.585-2.149	0.730	1.183	0.592-2.364	0.635
**PFS**						
**Treatment Group** CCRT+AC vs IC+ CCRT	1.199	0.621-2.313	0.589	1.214	0.625-2.357	0.568
**Age** ≥50 vs <50	1.414	0.758-2.636	0.276	1.338	0.692-2.588	0.386
**Sex** Male vs Female	0.578	0.297-1.125	0.107	0.539	0.274-1.061	0.073
**T classification** T3-4 vs T1-2	0.841	0.439-1.610	0.601	0.664	0.317-1.393	0.279
**N classification** N2-3 vs N0-1	0.650	0.296-1.427	0.283	0.568	0.229-1.405	0.221
**Stage** IVa/IVb vs III	1.099	0.574-2.106	0.776	1.199	0.599-2.399	0.609
**LRFS**						
**Treatment Group** CCRT+AC vs IC+ CCRT	1.268	0.657-2.449	0.479	1.293	0.665-2.511	0.449
**Age** ≥50 vs <50	1.484	0.794-2.773	0.216	1.408	0.725-2.733	0.312
**Sex** Male vs Female	0.564	0.290-1.099	0.092	0.523	0.266-1.031	0.061
**T classification** T3-4 vs T1-2	0.813	0.426-1.551	0.530	0.632	0.303-1.321	0.223
**N classification** N2-3 vs N0-1	0.650	0.296-1.428	0.283	0.559	0.224-1.392	0.212
**Stage** IVa/IVb vs III	1.106	0.577-2.119	0.762	1.216	0.608-2.433	0.580
**DMFS**						
**Treatment Group** CCRT+AC vs IC+ CCRT	1.096	0.568-2.115	0.784	1.087	0.559-2.114	0.807
**Age** ≥50 vs <50	1.439	0.772-2.684	0.252	1.396	0.718-2.714	0.326
**Sex** Male vs Female	0.579	0.298-1.128	0.108	0.534	0.270-1.056	0.071
**T classification** T3-4 vs T1-2	0.879	0.459-1.681	0.696	0.697	0.331-1.457	0.335
**N classification** N2-3 vs N0-1	0.658	0.300-1.446	0.298	0.603	0.243-1.496	0.275
**Stage** IVa/IVb vs III	1.110	0.579-2.128	0.752	1.173	0.586-2.349	0.653

^‡^P<0.05.

**Figure 3:**
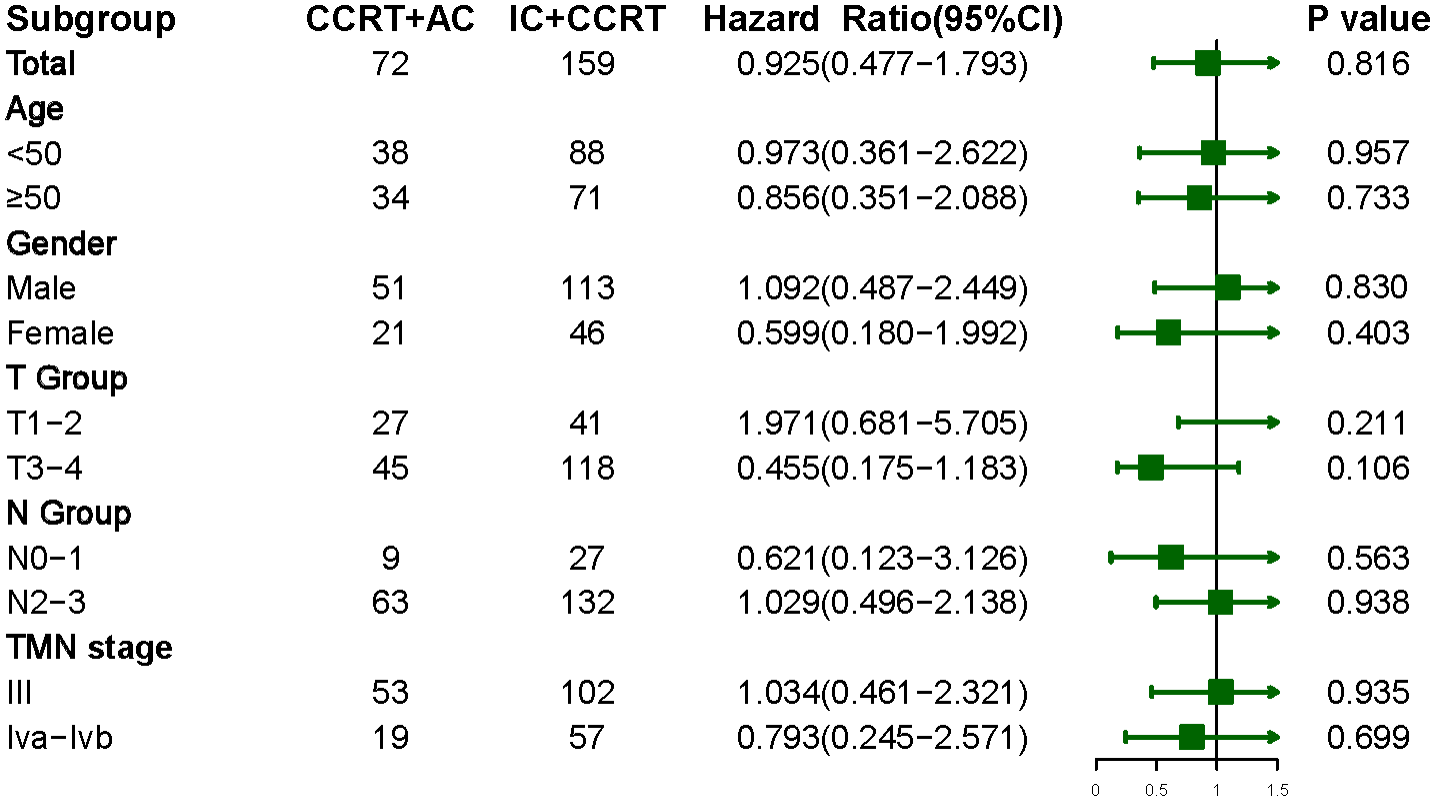
Overall survival in LANPC patients treated with IC+CCRT or CCRT+AC, stratified by age, gender, and tumor stage.

As shown in Figure 3, patient OS was stratified by age, gender, T classification, N classification, and clinical stage. Neither IC+CCRT nor CCRT+AC significantly differed in OS in each subgroup.

### Adverse events

Table 4 lists the grade 3-4 toxicities during treatment. In IC+CCRT, the most common grade 3-4 hematologic toxicity is neutropenia. It was also the most common hematologic adverse reaction of CCRT+AC. In comparison to patients treated with IC+CCRT, those treated with CCRT+AC had a significantly higher incidence of grade 3 to 4 leucopenia (p=0.001) and neutropenia (p=0.001). There was no difference in the incidence of thrombocytopenia, anemia, nausea, vomiting, or hepatoxicity between the two groups. (all p>0.05).

**Table 4:** Grade 3-4 toxicities during IC+CCRT or CCRT+AC in LANPC patients.

**Toxicities**	**Total N=231**	**IC+CCRT N=159**	**CCRT+AC N=72**	**P value^§^**
Neutropenia	50(21.6%)	25(15.7%)	25(34.7%)	0.001^*^
Leucopenia	49(21.2%)	24(15.1%)	25(34.7%)	0.001^*^
Thrombocytopenia	14(6.1%)	8(5.0%)	6(8.3%)	0.330
Anemia	13(5.6%)	8(5.0%)	5(6.9%)	0.559
Nausea	26(11.3%)	19(11.9%)	7(9.7%)	0.619
Vomiting	26(11.3%)	19(11.9%)	7(9.7%)	0.619
Hepatoxicity	2(0.9%)	2(1.3%)	0(0)	0.339

^§^P<0.05.

## DISCUSSION

For LANPC patients, IC plus CCRT and CCRT plus AC are both recommended treatment options. Additional chemotherapy paired with CCRT is regarded as a good therapeutic option for patients with LANPC. However, it is unclear whether additional chemotherapy should be given to these patients before or after concurrent systemic therapy/RT. In this study, we analyzed and compared the efficacy and toxicities between IC+CCRT and CCRT+AC for treating advanced NPC in the real world. According to our findings, IC+CCRT provided comparable survival benefits to CCRT+AC, while the latter increased treatment-associated toxicity.

Previous studies have demonstrated that CCRT combined with AC improves survival in patients with NPC compared to RT alone. However, further analysis suggests that the survival benefit is more likely to come from CCRT [12], and the value of AC is uncertain. In 2012, Chen et al. conducted a phase III clinical trial comparing adjuvant PF chemotherapy after CCRT with CCRT alone for LANPC. In the CCRT plus AC group, the predicted 2-year failure-free survival rate was 86%, compared to 84% in the CCRT group (HR= 0.74, 95% CI: 0.49–1.10; p=0.13) [13]. Furthermore, long-term follow-up results showed that AC did not provide any significant survival benefits [14]. However, in this study, only approximately 60% of the patients completed three cycles of AC. In addition, 50% of the patients required a reduced dosage due to intolerable side effects. Whether the decrease in the dose intensity of AC affects the survival benefit needs to be further verified, and some related retrospective analyses found that AC did not improve the outcomes of patients with LANPC [15, 16]. AC prolongs the treatment time and imposes additional toxicity and economic burden. In addition, considering that the compliance with AC was poor, only approximately 50-75% of patients completed the entire treatment [17], which also affected the efficacy of AC to a certain extent.

However, recent meta-analysis results showed that among the different combination modes of chemoradiotherapy, CCRT combined with AC still has the highest probability of improving tumor-related outcomes [10, 18]. Therefore, AC may still have a unique value. Due to patients' limited tolerance for AC and the uncertainties surrounding its efficacy, IC is thought to be a more practical and successful intense therapy approach; the target area of RT can be reduced by IC, subclinical metastases can be removed, and tumor lesions can be decreased. In addition, because it is carried out before CCRT, the general condition of patients is better, and they can tolerate the chemotherapy better [17, 19]. Sun et al. led a multinational phase III clinical trial on patients with stage III-IVb LANPC in 2016 [11]. In this study, 241 patients received TPF (docetaxel + cisplatin + fluorouracil) IC combined with three cycles of high-dose cisplatin (^100 mg/m2^) CCRT, and 239 patients received three cycles of cisplatin CCRT. After a median follow-up of 45 months, the 3-year failure-free survival was 80% in the IC plus CCRT group and 72% in the CCRT alone group (HR= 0.68, 95% CI: 0.48–0.97; p=0.034). This study was the first to confirm that based on IMRT plus concurrent chemotherapy, patients with LANPC may benefit from IC in terms of DFS and OS, as well as in a reduction in the occurrence of distant metastases. Moreover, in this investigation, the incidences of hematological toxicities, particularly neutropenia, were lower than the 55%-83% reported in earlier studies [20, 21]. Another clinical trial also found that the addition of GP (gemcitabine, cisplatin) to CCRT could improve OS and PFS when compared to CCRT alone [22]. Not only does it show advantages compared to CCRT, but among IC+CCRT, CCRT, CCRT+AC, IC+RT, and RT alone, a meta-analysis including only randomized controlled trials found that IC+CCRT may be the best option for the treatment of LANPC. Although this conclusion came from an indirect comparison, IC+CCRT outperformed CCRT in terms of survival benefit, but CCRT+AC was not superior to CCRT alone in regards of survival benefit [23].

In conclusion, both therapy modalities have advantages and downsides. Although the Intergroup-0099 trial demonstrated that CCRT plus AC improved 3-year OS, most patients experienced such severe side effects during CCRT that they could not tolerate additional AC treatment [9]. IC+CCRT seems to have better compliance and could eliminate micrometastasis. However, the lengthier waiting period before radiation may be deleterious to survival, and the side effects caused by IC prevent standard CCRT from being performed.

To reduce the potential confounding factors, we simulated the matching observed in randomized controlled trials. We used the PSM method to balance the baseline characteristics. Finally, 231 individuals with LANPC were included in this study, with 159 receiving IC+CCRT and 72 receiving CCRT+AC. After analysis, we found that IC plus CCRT offered higher 3- and 5-year OS rates than CCRT plus AC, but there was no significant difference. Moreover, other clinical outcomes (including PFS, LRFS, and DMFS) did not show a significant difference between the two groups at 3 or 5 years (p>0.05).

To some extent, this finding is consistent with the results of previous retrospective studies, which demonstrated that the outcome of IC+CCRT-treated patients was not superior to that of CCRT+AC [24, 25]. Setakornnukul et al. conducted a retrospective study on 266 patients, among whom 79 received IC+CCRT and 187 received CCRT+AC. Their study demonstrated that the 3-year and 5-year OS of LANPC patients treated with IC+CCRT were not better than that of CCRT+AC. However, after statistical correction for clinical stage, IC+CCRT revealed greater 3- and 5-year OS benefits than CCRT+AC, although the difference was not statistically significant [26]. Selection bias may be the underlying reason for this. It is worth mentioning that in our research, IC+CCRT also showed non-statistically significant superior 3- and 5-year OS rates. Another retrospective study of 550 patients with LANPC obtained the same results, no difference in 3- and 5-year OS, DMFS, LRFS, or PFS between IC+CCRT and CCRT+AC groups [25]. Our results are partially consistent with the aforementioned findings; however, its subgroup analysis showed that IC+CCRT was associated with improved survival compared to CCRT+AC in T3 or N2 NPC patients. Another related study pointed out that the treatment group (IC-CCRT vs. CCRT-AC) was an independent predictive factor for survival; IC-CCRT was preferable in stage III disease, but CCRT-AC may be more beneficial for people with stage IVa disease [27]. However, in our subgroup analysis, there was no significant survival difference between patients treated by IC+CCRT or CCRT+AC, and the difference may be attributed to the limited sample size and different selection criteria.

In the absence of randomized clinical trials, there are no direct comparisons of the adverse reactions between the two treatments. However, the results of a meta-analysis showed that as measured by p-scores, CCRT+AC and RT+AC had the most negative responses among all available treatments (including IC+CCRT and CCRT+AC) [10], highlighting the potential toxicity of AC. Similar to the findings of this study, our research found that patients treated with CCRT+AC had a higher incidence of grade 3 to 4 leucopenia (p=0.002) and neutropenia (p=0.003) than those treated with IC+CCRT. In summary, we conclude that IC+CCRT and CCRT+AC have equivalent treatment efficacy for stage III-IVb NPC patients. However, considering patient compliance and adverse effects, IC+CCRT may be a more viable treatment option.

This research had several strengths. First, we collected patients' adverse reactions compared with those in previous retrospective studies. We analyzed the toxicities between the two groups. The second advantage of our study is that we collected data from two different research institutions in Sichuan and Beijing, enhancing its authenticity and reliability.

Our study has some limitations. First, this study is a retrospective analysis, inevitably with internal bias. The chemotherapy regimens of AC and IC were not wholly consistent due to the actual clinical situation. Second, some common factors affecting prognosis, such as EBV-DNA, were not included in the research because of the large amount of missing data.

## CONCLUSIONS

Compared with CCRT+AC, IC+CCRT had a comparable survival benefit and a lower frequency of treatment-related adverse events. IC+CCRT may be a promising therapeutic strategy based on its relative efficacy and side effects. Prospective investigations are required to confirm this finding.

## MATERIALS AND METHODS

### Patients

Patients with LANPC were recruited from two Chinese medical centers between January 2009 and May 2020. The inclusion criteria were listed below: (a) Pathological diagnosis of nasopharyngeal carcinoma. (b) Clinical stage III-IVb according to the 7th edition of the American Joint Committee on Cancer (AJCC) staging criteria. (c). Receiving either IC plus CCRT or CCRT plus AC, in which RT was based on IMRT. (d) Patients with complete follow-up information. The following were the exclusion criteria: (a) clinical stage I or II or IVC and (b) People having a prior history of malignancy or a second primary tumor.

### Treatment

Patients were given IMRT for primary tumor site and involved lymph nodes. Briefly, planned target volume (PTV) dose was 70-74 Gy for the primary gross tumor volume (GTVnx), 70 Gy for the metastatic lymph nodes (GTVnd), and 60 Gy for the CTV1 volume (high-risk clinical tumor volume). CTV1 was defined as the GTV plus a margin of 5-10 mm, and it included the entire nasopharyngeal mucosa as well as a 5-mm submucosal block; CTV2 requires 54-56 Gy (low-risk clinical target volume), which included suspected subclinical sites of spread. For a total of 33-35 fractions, each patient received radiation therapy once a day, Monday through Friday.

Chemotherapy regimens in CCRT mainly included cisplatin (75-100 mg/m^2^ every 3 weeks). IC regimens mainly included TPF (docetaxel 60 mg/m^2^ on Day 1, cisplatin 60 mg/m^2^ on Day 1, 5-fluorouracil 600 mg/m^2^ on Day 1-5, every 3 weeks), GP (gemcitabine 1000 mg/m^2^ on Day 1 and 8, cisplatin 80 mg/m^2^ on Day 1, every 3 weeks), TP (docetaxel 75 mg/m^2^ on Day 1, cisplatin 75 mg/m^2^ on Day 1, every 3 weeks) and PF (100 mg/m^2^ cisplatin on Day 1 and 1000 mg/m^2^ 5-fluorouracil on Days 1-4, repeated every 3 weeks). AC regimens comprised TP (docetaxel 75 mg/m^2^ on Day 1, cisplatin 75 mg/m^2^ on Day 1, every 3 weeks) and PF (100 mg/m^2^ cisplatin on Day 1 and 1000 mg/m^2^ 5-fluorouracil on Days 1-4, repeated every 3 weeks).

### Covariates and matching

PSM was used to balance the covariates between the two treatment groups. We performed a logistic regression analysis using the characteristic data to identify the correlation between each factor and the chosen treatment. The traits that affected treatment selection served as the independent variables in this regression, while the treatment modalities served as the binary dependent variable. Based on each subject's unique characteristics, a predicted probability of treatment, ranging from 0 to 1, was calculated using the coefficient estimates from this regression. Then, depending on the size of the available control group, each patient in the IC+CCRT group was matched to one or more patients in the CCRT+AC group. All matched treatment units and control units were then analyzed for differences in the results, and all unmatched control units were removed.

### Endpoint definition

Our study's primary outcome was OS, defined as the time between the initial diagnosis and death due to any cause. Secondary study endpoints included progression-free survival (PFS), locoregional recurrence-free survival (LRFS), distant metastasis-free survival (DMFS), and side effects. PFS was defined as the treatment date until disease progression or death due to any reason. LRFS was defined as the time until locoregional recurrence (primary tumor or lymph nodes) occurred. DMFS was defined as the treatment date for distant metastasis. The Common Terminology Criteria for Adverse Events (CTCAE 4.0) were used to assess treatment-related toxicity.

### Statistical analysis

As a precaution to eliminate covariate imbalances in the cohort, we used PSM models with calipers of 0.02 to balance the variables, thus approximating a randomized experiment in an observational study. The chi-square test was employed to compare the baseline characteristics and toxic effects between the two groups. Clinical outcomes, such as OS, PFS, LRFS, and DMFS, were analyzed using Kaplan-Meier curves and log-rank tests. Univariate and multivariate Cox proportional hazard analyses were performed to identify independent prognostic factors. R (version 3.62) and IBM SPSS 25.0 (Armonk, NY, USA) were used for the statistical analysis. All statistical tests were two-sided, with a p-value <0.05 considered significant.

## Supplementary Material

Supplementary Figure 1
