# Supplementary material for: Induction versus adjuvant chemotherapy combined with concurrent chemoradiotherapy in locoregionally advanced nasopharyngeal carcinoma: a retrospective cohort study
Source: Aging (Albany NY). 2022 Aug 26;14(16):6727–39. doi: 10.18632/aging.204246 (PMC9467407; doi:10.18632/aging.204246)
Supplement: Supplementary Figure 1 [file aging-14-204246-s001.pdf]

SUPPLEMENTARY FIGURE

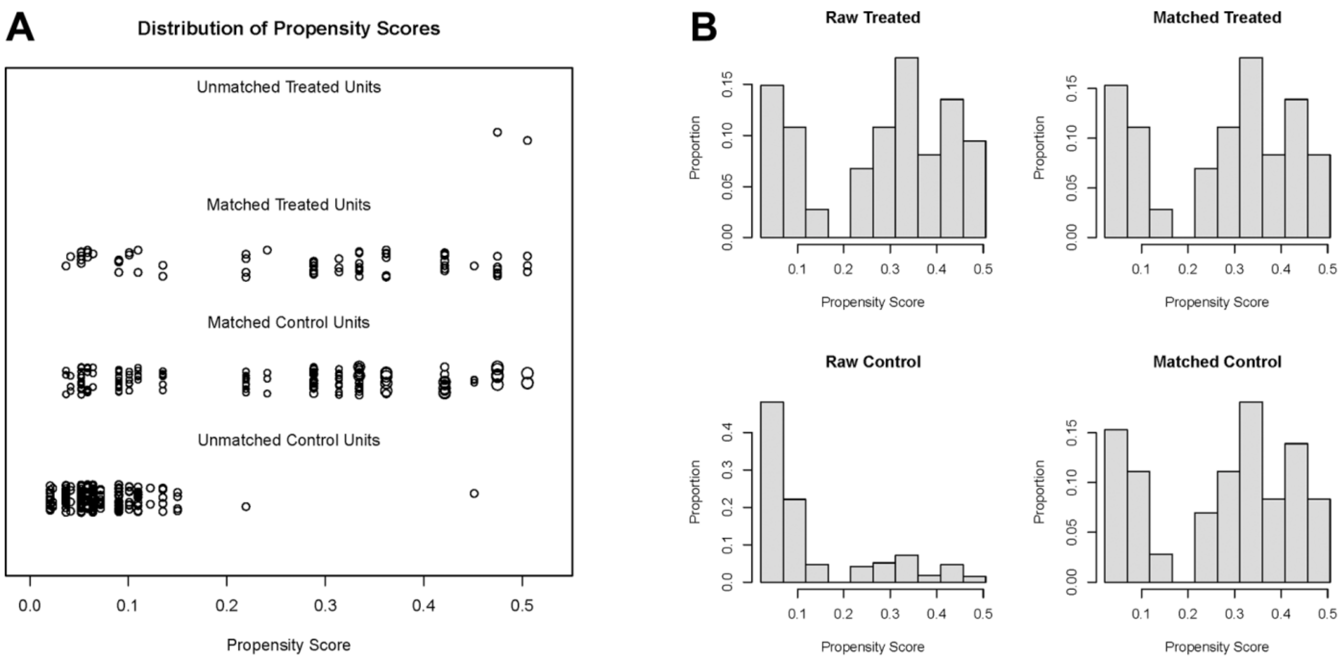

Supplementary Figure 1. (**A**) Distribution of propensity scores between IC+CCRT and CCRT+AC groups; (**B**) Histogram of propensity scores in the raw and matched groups.
